# Prediction of hereditary angioedema during attacks in patients with recurrent angioedema: Awareness at a glance with the hereditary angioedema prediction score

**DOI:** 10.1002/clt2.70040

**Published:** 2025-04-16

**Authors:** Semra Demir, Müge Olgaç, Osman Ozan Yeğit, İlkim Deniz Toprak, Mehmet Erdem Çakmak, Merve İğde Hormet, Nida Öztop, Pelin Korkmaz, Şule Kamacı Çelik, Deniz Eyice Karabacak, Nevzat Kahveci, Işıl Göğem İmren, Bircan Erden, Raif Coşkun, Pelin Karadağ, Derya Ünal, Aslı Gelincik

**Affiliations:** ^1^ Immunology and Allergy Division Internal Medicine Department Istanbul Faculty of Medicine Istanbul University Istanbul Turkey; ^2^ Adult Immunology and Allergy Clinic Seyrantepe Hamidiye Etfal Research and Training Hospital Istanbul Turkey; ^3^ Adult Immunology and Allergy Clinic Başakşehir Pine and Sakura City Hospital Istanbul Turkey; ^4^ Adult Immunology and Allergy Clinic Prof. Dr. Cemil Taşçıoğlu City Hospital Istanbul Turkey

**Keywords:** angioedema, awareness of hereditary angioedema, hereditary angioedema, prediction of hereditary angioedema, recurrent angioedema

## Abstract

**Background/Aim:**

Misdiagnosis of hereditary angioedema (HAE) leads to inappropriate management of the attacks. A scoring system that anticipates diagnosis can be beneficial for clinicians who are unfamiliar with angioedema. This study aims to develop a practical scoring system for use during acute attacks to predict HAE in patients with recurrent angioedema (RAE).

**Method:**

To predict HAE, nine HAE experts unanimously identified five predictive items (PIs); absence of urticaria, presence of abdominal pain episodes, family history, early onset of attacks and previous unresponsiveness to anti‐histaminergic treatments. The researchers questioned 106 patients with HAE and 155 patients with mast cell‐mediated angioedema (MMAE) about PIs. A score was attributed to each significant PI based on OR values obtained through logistic regression analysis. The cut‐off point for the prediction of HAE and its sensitivity and specificity were determined by ROC curve analysis.

**Results:**

In a univariate analysis, all items showed significant differences between HAE and MMAE patients. Regression analysis attributed scores as follows: 23 points for the absence of urticaria, 11 points for the abdominal pain episodes, 9 points for family history, and 53 points for unresponsiveness to antihistaminergic treatments. No score was attributed to early onset of age (*p* > 0.05). The ROC analysis revealed an area under the curve of 0.990, with a total score of ≥38 demonstrating the best sensitivity (96.4%) and specificity (96.1%).

**Conclusions:**

HAEps is a valuable tool for diagnosing HAE in patients with RAE. A score of 38 or more indicates the possible presence of HAE with substantial sensitivity and specificity.

## 1 INTRODUCTION

Recurrent angioedema (RAE) that occurs more than once can be caused by various mechanisms such as bradykinin or mast cell mediated or intrinsic vascular endothelium dysfunction.^1^ Accurate diagnosis is essential for patients with RAE as the treatment approach varies depending on the type of AE. Hereditary or acquired factors can lead to development of bradykinin mediated angioedema (BMAE). Medications such as angiotensin‐converting enzyme inhibitors (ACEI), dipeptidyl peptidase IV (DPPIV) inhibitors, or underlying autoimmune or myeloproliferative disorders can cause acquired RAE episodes of BMAE, which can be prevented by avoiding the medications or treating the underlying diseases. Hereditary forms include hereditary angioedema (HAE) with C1 inhibitor deficiency and HAE with normal C1 inhibitor.^2^ HAE is a rare, life‐threatening and autosomal dominantly inherited disease that results in unpredictable recurrent swelling episodes of the face, airways, genitals, extremities and abdomen. The deficiency in the level or function of C1 inhibitor caused by the mutations in the SERPING1 gene leads to the majority of the HAE types 1 and 2, respectively.^3^ The defect in the C1 inhibitor leads to increased serum bradykinin levels. These elevated bradykinin levels bind to the bradykinin receptor 2, causing increased vascular permeability and fluid leakage from blood vessels and tissue.^4^, ^5^

HAE is characterized by recurrent episodes of well‐demarcated, nonitchy swelling and/or abdominal pain. The severity and frequency of these attacks can vary significantly between patients and even within the same individual. Typically, the first symptoms appear around the ages of 11–12, and the attacks usually resolve within 2–5 days.^6^, ^7^ While physical or emotional trauma and hormonal changes can trigger HAE attacks in some patients, they can also occur spontaneously.^2^

A major challenge in managing HAE is the high rate of misdiagnosis and delayed diagnosis, which can leave many patients without proper treatment. The average time between symptom onset and an accurate diagnosis was reported as 17 years in Türkiye, 16 years in China, and between 8 and 21 years for HAE Types 1 and 2 in Europe.^7^, ^8^, ^9^ However, a recently published study from Europe indicated that this timeframe has been decreasing to approximately 2 years for patients with a family history and 6 years for those without.^10^ Abdominal swelling episodes can be extremely painful and, in some cases, lead to unnecessary surgery or hypovolemic shock due to the massive fluid shifts. Additionally, over half of HAE patients will experience at least one episode of potentially fatal laryngeal edema during their lifetime, with an asphyxiation mortality risk of 14%–33% in untreated cases.^3^

Effective HAE management involves avoiding known triggers, preventing recurrent attacks, and promptly treating acute episodes. Medications such as C1 inhibitor concentrates, bradykinin receptor antagonists (e.g., icatibant), and kallikrein inhibitors (e.g., ecallantide) are the primary treatment options for managing HAE attacks.^2^

Mast cell‐mediated angioedema (MMAE) usually accompanies pruritic wheals in patients with chronic spontaneous urticaria (CSU). Antihistamines, corticosteroids and/or epinephrine are used for the treatment of MMAE attacks.^2^ Some patients with MMAE may experience isolated RAE.^11^, ^12^ Furthermore, CSU, being more prevalent, can also occur in patients with HAE.^13^, ^14^, ^15^ Therefore, the presence of urticaria does not exclude the possibility of coexisting HAE, while the absence of urticaria does not eliminate the possibility of MMAE.^2^ Patients with RAE often present to the emergency room during attacks. Unfortunately, due to misdiagnosis or an undiagnosed HAE, they frequently fail to receive appropriate treatment. Untreated, these debilitating or potentially fatal attacks impose a significant burden on patients and their families, including reduced quality of life, increased healthcare costs, and the risk of death or unnecessary surgeries.^3^, ^16^, ^17^ Therefore, recognizing potential HAEs in the emergency room is crucial for early intervention to reduce disease burden and prevent mortality and morbidity. Currently, there is only one published tool for predicting HAE attacks in the ER. The authors reported that if a patient with RAE has a history of recurrent abdominal pain and the attacks do not respond to allergy treatment, HAE should be considered and treatment with C1 inhibitor and/or icatibant and/or ecallantide should be initiated promptly.^18^ Additionally, a positive family history, upper airway edema, early onset of symptoms, prodromal signs and absence of pruritic wheals strongly suggest HAE type 1–2 as per international guideline.^2^

Therefore, we aimed to develop a practical scoring system, the hereditary angioedema prediction scoring system (HAEps), to assist clinicians who are unfamiliar with angioedema in predicting HAE during acute attacks.

## 2 METHODS

Nine HAE experts from four adult allergy centers including the Division of Adult Immunology and Allergy Diseases at Istanbul Faculty of Medicine, the Adult Allergy and Immunology Clinics at Seyrantepe Hamidiye Etfal Research and Training Hospital, Başakşehir Pine and Sakura City Hospital, and Prof. Dr. Cemil Taşçıoğlu City Hospital participated in the study. These experts who were trained in one of the angioedema referral centers and currently see the patients with RAE convened multiple times, both in person or online collaboratively to develop the scoring system.

The HAEps was developed through four main phases: generation of predictor items (PIs), reduction and selection of PIs, application of the scale to patients with HAE or MMAE and analysis of the items leading to generation of the scoring system. HAE type 1 and 2 were diagnosed based on recent guidelines (2) and MMAE was defined as the presence of recurrent episodes of sudden, noticeable swelling of the mucocutaneous tissues, with or without urticaria, that responds well to antihistamines, glucocorticoids, and/or epinephrine (11).

### 2.1 Phase 1: Generation of predictor item pool

The experts convened to establish the conceptual framework and to develop a prototype scale.^19^

A comprehensive literature review was conducted to document the HAE‐related features. Based on this review and their clinical experience, the experts identified a list of predictor items (PIs). A total of 46 questions were generated to assess about clinical features comprising onset age of disease, presence of urticaria and other symptoms related to mast cell degranulation, episodes of abdominal pain, usage of medications: ACEIs/Angiotensin receptor inhibitors (ANRIs)/DPPIV‐inhibitors, non‐steroidal anti‐inflammatory drugs (NSAIDs)/oral contraceptive pills (OCP), or others, presence of triggers (allergens, physical factors, trauma, stress), locations of AE, timing of AE development and resolution, prodromal symptoms, response to mast cell mediator targeted treatment including antihistamine, corticosteroids and or epineprine, and family history. Unresponsiveness to mast cell mediator‐targeted treatments, including antihistamines, corticosteroids, and/or epinephrine, was defined as ‘the absence of regression in the angioedema attack within 2 hours after treatment administration,’ based on our previously published data.^20^

### 2.2 Phase 2: Reduction and selection of the items

During expert meetings, the pre‐formulated questions were reassessed to achieve consensus on the final structure. Experts evaluated each item, categorizing them as either agreed or disagreed. Items that achieved at least 80% agreement were included in the final list of PIs otherwise were excluded (Table 1). Additionally, the experts unanimously decided to exclude patients who experienced the angioedema attacks with any triggers including medications, foods or venoms and who were receiving treatment with ACE inhibitors (ACEIs), angiotensin receptor‐neprilysin inhibitors (ARNIs), DPPIV inhibitors, NSAIDs or OCP. Conversely, they agreed to include patients who had previously visited the emergency room (ER) and had undergone mast cell mediator‐targeted (MCMT) treatments, as well as patients with confirmed diagnoses of MMAE and HAE type 1 and 2 otherwise were excluded.

**TABLE 1:** The final list of predictor items.

Predictor items	Agreement degree
Absence of urticaria	100%
Presence of abdominal pain episodes	100%
Early onset of attacks (<18 years of age)	88.8%
Unresponsiveness to MCMT treatments	100%
Family history	100%

Abbreviation: MCMT, mast cell mediators targeted.

### 2.3 Phase 3: Application of scale to the patients

In this phase, we administered the scale to patients with confirmed diagnosis of HAE type 1 and 2 as well as those with MMAE based on guidelines to collect data to evaluate the predictive capacity of the PIs included in the scale.^2^, ^12^ Patients were interviewed during their routine visits, following the acquisition of their written informed consent.

### 2.4 Phase 4: Data analysis and development of the HAEps scoring system

The HAEps, scoring system was developed through multivariate analysis to examine the relationship of each item with the presence of HAE.^21^, ^22^ Multicollinearity was assessed by the condition index. In the regression analysis, estimated risk values for related factors were rounded to the nearest number to serve as scores in the scoring system. The scoring system's cut‐off point for predicting the presence of HAE was determined using receiver operating characteristic (ROC) curve analysis. Additionally, the area under curve (AUC), along with sensitivity and specificity, were calculated for the system's effectiveness. The optimal cut‐off point was selected based on the Youden index.^23^

The study was conducted with the approval of the local Ethics Committee of Istanbul Faculty of Medicine.

### 2.5 Statistical analysis

The categorical variables were expressed as frequencies and percentages, while continuous variables were presented as medians with interquartile ranges 25–75 or mean ± standard deviation, depending on the distribution of data. The Pearson χ^2^ or Fisher's exact test for categorical variables and Mann‐Whitney U or Student's *t*‐test for numeric variables were used to compare where appropriate. The predictor items for the presence of HAE were assessed through univariate and multivariate binary logistic regression analyses, with results reported as odds ratio (OR) with a relevant 95% confidence interval (CI). Variables demonstrating a *p*‐value of less than 0.05 in the univariate analysis were further examined using multivariate analysis. Details regarding the scoring system generation can be found in the method section. The data were analyzed by IBM SPSS Statistics for Windows, Version 24.0 released in 2016 (Armonk, NY: IBM Corp.).

## 3 RESULTS

A total of 106 patients with confirmed HAE type 1 or 2 and 155 patients with a MMAE diagnosis were evaluated through questionnaires and medical record reviews. The median age of the patients was 41.5 (IQR = 29–51) years. Patients with MMAE were slightly older than those with HAE (*p* = 0.003) (Table 2). Among the patients, 68.4% of those with MMAE and 61.9% of those with HAE were female, with no statistically significant difference (*p* > 0.05) (Table 2). The median (IQR) age of onset of symptoms was 36 (25–45) for MMAE patients and 13 years (IQR = 7–19.25 years) for HAE patients.

**TABLE 2:** Comparison of patients with MMAE and HAE by univariate analysis.

	MMAE (*n* = 155)	HAE (*n* = 106)	*p*
Age (year), median (IQR)	43 (51–32)	35 (24–47.5)	0.003
Gender			
Female, *n* (%)	106 (68.4)	65 (61.9)	
Male, *n* (%)	49 (31.6)	40 (38.1)	>0.05
Absence of urticaria, *n* (%)	40 (25.8)	100 (94.3)	<0.001
Presence of abdominal pain episodes, *n* (%)	15 (9.8)	83 (78.3)	<0.001
Presence of family history, *n* (%)	17 (11)	85 (80.2)	<0.001
Early onset of symptoms (≤18 years of age), *n* (%)	8 (5.2)	77 (72.6)	<0.001
Unresponsiveness to MCMT treatments, *n* (%)	6 (3.9)	97 (91.7)	<0.001

Abbreviations: HAE, hereditary angioedema; IQR, interquartile range; MCMT, mast cell mediators targeted; MMAE, mast cell‐mediated angioedema.

In a univariate comparative analysis, patients with HAE showed a higher frequency of the absence of urticaria, past abdominal pain attacks, a family history of the condition, early onset of symptoms, and unresponsiveness to MCMT treatments compared with those with MMAE (*p* < 0.001 for each factor) (Table 2).

### 3.1 Generation of the HAEps scoring system

To develop a prediction scoring system for HAE, logistic regression analysis was performed using predictor items that were significantly associated with the presence of HAE in univariate analysis. These predictors included absence of urticaria, presence of abdominal pain, early onset of symptoms, unresponsiveness to MCMT and presence of family history (Table 2). The OR values were rounded to the closest decimal to obtain a score for each PI. In the multivariate analysis, absence of urticaria, presence of abdominal pain, unresponsiveness to MCMT treatments, and presence of family history were closely related to HAE (Table 3). Accordingly, the scoring system assigned points as follows: absence of urticaria 23 points, presence of abdominal pain 11 points, presence of family history 9 points, and unresponsiveness to MCMT treatments 53 points and the total score range from 0 to 96 (Table 3).

**TABLE 3:** Multivariate analysis of predictive items and accordingly determined scores.

	MMAE (*n* = 155)	HAE (*n* = 106)	*p*	OR (CI 95%)	Score
Absence of urticaria, %	25.8	94.3	0.004	23.09	23
Presence of abdominal pain episodes, %	9.8	78.3	0.012	11.38	11
Presence of family history, %	11	80.2	0.039	8.91	9
Early onset of symptoms (≤18 years of age), %	5.2	72.6	0.097	‐	‐
Unresponsiveness to MCMT treatments, %	3.9	91.7	<0.001	53.35	53

Abbreviations: HAE, hereditary angioedema; MCMT, mast cell mediators targeted; MMAE, mast cell‐mediated angioedema.

**FIGURE 1:**
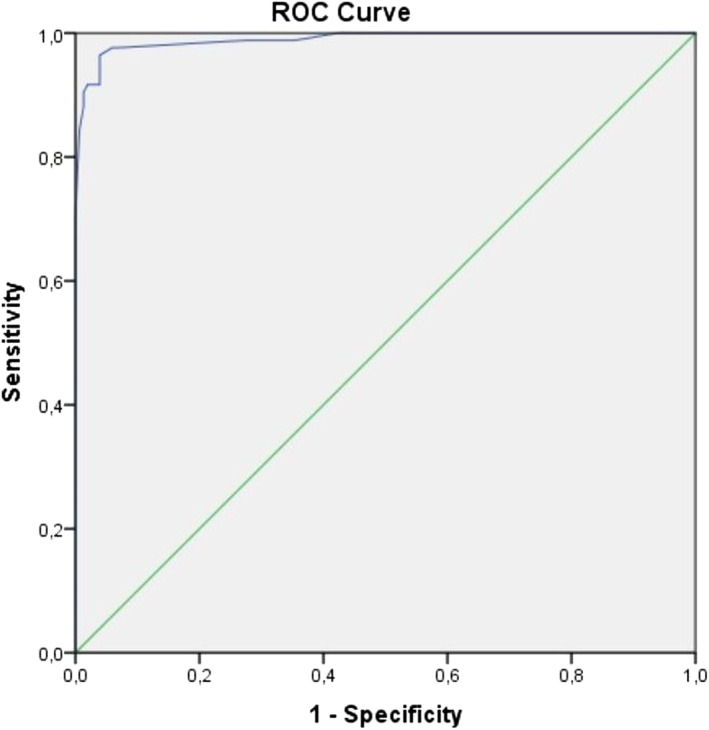
ROC curve analysis of HAEps scores (AUC = 0.990, CI 95% = 0.979–1). ROC, receiver operating characteristic.

The scores for each patient were calculated, and the ROC curve analysis was performed to determine a cut‐off point for predicting HAE. The AUC was 0.990, (CI 95% = 0.979–1) (Figure 1). A score of 38 points or more indicated the presence of HAE with a 96.4% sensitivity and 96.1% specificity (Table 4).

**TABLE 4:** Sensitivity and specificity values of cut‐off points obtained from ROC curve analysis.

Cut‐off points	Sensitivity	Specificity
≥10	98.8%	64.7%
≥15	98.8%	70.6%
≥21	98.8%	72.5%
≥27	97.6%	93.5%
≥33	97.6%	94.1%
≥38	96.4%	96.1%
≥48	91.7%	96.1%
≥57	91.7%	98%

## 4 DISCUSSION

The current study presents a predictive scoring system designed to identify HAE types 1 and 2 primarily, in patients with RAE. This system demonstrates substantial sensitivity and specificity in addressing a critical need in clinical practice. By facilitating prompt and accurate treatment, particularly in emergency settings, the scoring system can significantly benefit patients. Furthermore, it aids in diagnosing previously undiagnosed individuals, allowing for the implementation of appropriate treatment plans. This proactive approach has the potential to reduce the frequency of attacks, minimize emergency admissions, lower healthcare costs, and improve the quality of life for those affected by angioedema attacks.^16^, ^17^, ^18^, ^24^

RAE is a significant cause of ER visits and more than one million people with this disease visit the ER each year.^25^, ^26^ However, a recently published cohort study including the 485 patients with HAE type 1 and 2 analyzed the specialties of physicians who diagnosed HAE in these patients. They reported that physicians working in ER diagnosed only 2.5% of them.^27^ Furthermore, reports indicated that the fatal laryngeal episodes and asphyxiation can occur in patients with HAE within 15 min.^28^

Given the severity of angioedema episodes, which can lead to death or debilitating consequences, prompt treatment is crucial in order to prevent adverse outcomes. AE is mediated by various mechanisms including mast cell mediators and bradykinin, which influence treatment options. Therefore, accurate diagnosis of the type of angioedema is essential for appropriate management.^29^

Patients who present with AE attacks without a definitive diagnosis can pose challenges for clinicians as some may lack awareness of the different causes and may not know how to differentiate or treat the various types of angioedema.^7^, ^30^, ^31^ Consequently, a practical tool for identifying the different types of angioedema and guiding appropriate treatment would be highly beneficial for clinicians, particularly those working in emergency settings.

In 2017, a review paper was published to assist the clinicians in differentiating between types of AE.^31^ The authors proposed an algorithm indicating that in patients with AE, the absence of urticaria or multisystem involvement such as anaphylaxis, ACE inhibitor or NSAID usage, or known allergens along with a family history, should prompt consideration of HAE. They also highlighted that the presence of abdominal pain, unresponsiveness to antihistaminic treatments, and a slow onset of attacks may suggest HAE.^31^ However, it is important to note that these recommendations were based solely on a review and lacked statistical validation.

In 2019, a group of authors introduced a rapid triage tool designed to predict HAE in ER. The experts agreed to include four key items: recurrent abdominal pain, absence of urticaria, family history and unresponsiveness to allergy treatment. These criteria were tested through retrospective chart reviews of 66 patients with HAE and 41 patients without HAE. The analysis concluded that if a patient with RAE has a history of abdominal pain episodes and shows unresponsiveness to allergy treatment, HAE should be considered, allowing for treatment with C1 inhibitor extract, icatibant, or fresh frozen plasma. This protocol, known as the HAE Rapid Triage Tool (HAE‐RT), demonstrated an impressive 98% sensitivity and specificity.^18^ In contrast to this model, our current study revealed that the absence of urticaria and presence of a family history also have a statistically significant relationship with the likelihood of HAE. Interestingly, the HAE‐RT study reported a 29% frequency of urticaria in patients with HAE, which was higher than expected.

The authors suggested that this discrepancy might stem from the misinterpretation of an essential prodromal symptom of HAE, erythema marginatum. Additionally, while family history is not mandatory for diagnosing HAE, our scoring system underscored its importance in differentiating HAE, even though it was not found to be significant in the HAE‐RT study.

Our study encountered some limitations. First of all, HAE with normal C1 inhibitor or acquired C1 inhibitor deficiency are extremely rare, and their clinical features are not as well‐defined as those of HAE type 1 and 2; they are also heterogeneous. Consequently, we excluded these patients to avoid potential challenges in differentiation. Our primary focus was to identify the more common forms of HAE. We believe that our model may have the potential to detect HAE with normal C1 inhibitor and acquired C1 inhibitor deficiency, but advanced assessments are necessary for confirmation. Secondly, this study was conducted exclusively with patients who had a confirmed diagnosis. Therefore, the HAE patient scores (HAEps) need to be validated in a prospective study that includes undiagnosed patients with recurrent angioedema (RAE) who present to the emergency room during acute attacks; this will be the focus of our next research initiative. Furthermore, the study included patients from a single country, Türkiye. Although there are no known ethnic differences in the features of HAE, this could present a potential limitation, highlighting the need for model validation. Lastly, the scoring system utilizes two‐digit numbers, which can complicate mental calculations. We believe that this scoring method could be effectively adapted into an artificial intelligence application or a web‐based calculator for easier use.

## 5 CONCLUSION

In this study, we developed a predictive scoring system called HAEps to diagnose HAE in patients with RAE admitted to the emergency room. A score of 38 points or higher indicated the presence of HAE with high sensitivity and specificity. This tool can help physicians predict HAE in undiagnosed patients, enabling prompt treatment, reducing hospitalizations, healthcare costs, and improving patients' quality of life.

However, our findings need validation through a comprehensive multicenter study. We believe that adapting HAEps to artificial intelligence would make it highly practical and useful in clinical settings.

## AUTHOR CONTRIBUTIONS

Semra Demir, Müge Olgaç, Osman Ozan Yeğit, İlkim Deniz Toprak, Mehmet Erdem Çakmak, Merve İğde Hörmet, Pelin Korkmaz, Nida Öztop, Şule Kamacı Çelik, Deniz Eyice Karabacak, Derya Ünal, Işıl Göğem İmren, Nevzat Kahveci, Bircan Erden, Raif Coşkun, Pelin Karadağ and Aslı Gelincik made substantial contributions to conception and design, acquisition of data, and analysis and interpretation of data; and were involved in drafting the manuscript or revising it critically for important intellectual content; and gave final approval of the version to be published. Each author participated sufficiently in the work to take public responsibility for appropriate portions of the content; and agreed to be accountable for all aspects of the work in ensuring that questions related to the accuracy or integrity of any part of the work are appropriately investigated and resolved.

## CONFLICT OF INTEREST STATEMENT

The authors declare no conflicts of interest.
